# Positive pathogens in stool could predict the clinical outcomes of sepsis-associated acute kidney injury in critical ill patient

**DOI:** 10.1038/s41598-024-62136-6

**Published:** 2024-05-16

**Authors:** Yaoyuan Cao, Fuxing Deng

**Affiliations:** 1 Department of Forensic Medicine, School of Basic Medical Sciences, Central South University, No 172. Tongzipo Road, Changsha, 410013 Hunan People’s Republic of China; 2 National Clinical Research Center for Geriatric Disorders, Xiangya Hospital, Central South University, Changsha, 410008 China

**Keywords:** Acute kidney injury, Bacterial infection, Stool pathogens, *Clostridium difficile*, Acute kidney injury, Risk factors, Bacterial infection, Clostridium difficile, Outcomes research

## Abstract

In this study, we sought to evaluate the influence of positive pathogens in stool (PPS) on clinical outcomes in critical ill patients with Sepsis-associated acute kidney injury (S-AKI) from intensive care unit. Our sample consisted of 7338 patients, of whom 752 (10.25%) had PPS. We found that the presence of *Clostridium difficile* (*C. difficile*) and protists in stool samples was correlated with survival during hospitalization, as well as 30-day and 90-day survival. Interestingly, there was no significant difference in overall survival and 30-day in-hospital survival between the PPS group and the negative pathogens in stool (NPS) control group. However, the cumulative incidence of 90-day infection-related mortality was significantly higher in the PPS group (53 vs. 48%, *P* = 0.022), particularly in patients with *C. difficile* in their stool specimens. After adjusting for propensity scores, the results also have statistical significance. These findings suggest that PPS may affect the 90-days survival outcomes of S-AKI, particularly in patients with *C. difficile* and protists in their stool samples. Further research is warranted to further explore these associations.

## Introduction

Sepsis association acute kidney injury (AKI) is a multifactorial clinical syndrome and a serious complication in critically ill patients^1–3^. It is associated with high mortality and increased healthcare costs. The mortality rate among AKI patients receiving renal replacement therapy (RRT) exceeds 50%, and the pathogenesis of this condition is not fully understood^4,5^. Sepsis is the leading cause of AKI in the intensive care unit (ICU). It can result from the destruction of the patient's mucosal barrier, an imbalance in the intestinal flora, the use of antibiotics and a lack of intestinal nutrition^6,7^. These factors can induce inflammatory responses that compromise host metabolism and immunity and promote the development of AKI. Several studies have demonstrated an association between gut microbiota and kidney injury, suggesting a close link between the kidneys and the gastrointestinal tract, known as the gut-kidney axis^8–10^. Dysregulated interactions between the intestinal epithelium, immune system, endogenous pathogens and kidneys may exacerbate systemic inflammation and AKI. Therefore, the relationship between stool pathogens and sepsis association AKI is of great importance. It can guide the subsequent treatment of patients with kidney disease and help reverse gastrointestinal dysfunction.

The gut-kidney axis has received considerable attention in recent years due to the intriguing interactions between these two organ systems. Acute kidney damage can be caused by pathogens in the intestines^11^. Several studies have investigated the impact of the stool pathogens on the progression of sepsis association AKI to chronic kidney disease (CKD)^12–15^. Given that most patients with sepsis are routinely treated with antibiotics, this could potentially lead to an imbalance in the intestinal flora and result in dynamic changes in the bacterial flora. A positive pathogen in stool (PPS) may reflect the dominant microorganisms in the gastrointestinal tract. The stool pathogens of patients with treated sepsis association AKI may be altered during transplantation due to intensive conditioning and administration of therapeutic antibiotics^16^.

However, the role of stool pathogens in sepsis-associated AKI has not been established by studies. The aim of this retrospective study was to determine the impact of stool pathogens on clinical outcomes in patients with sepsis-associated AKI. To control for confounders, we also performed propensity score matching (PSM) and causal inference for the analysis.

## Results

### Results of stool cultures

A total of 27,234 stool samples were collected from 7338 patients. A PPS was observed in 752 patients (10.25%), whose age, weight, and sex were comparable to those of the persistently negative pathogens in stool (NPS) group (Table 1). The most common pathogen identified in stool samples was *C. difficile*, followed by *Adenovirus*, *Enterococcus sp.*, and *Salmonella species* (Table 2). One patient’s staphylococcal sample contained *Methicillin-resistant Staphylococcus*. The most common virus identified from stool samples was *Adenovirus*, while the most common Enterobacteriaceae was *Salmonella*. Multiple pathogens (≥ 2) were detected in 4 patients.

**Table 1:** Baseline characteristics between the two groups positive pathogens in stool and negative pathogens in stool.

Variables	Total (n = 7338)	Stool negative (n = 6586)	Stool positive (n = 752)	*P* value
Age, median (Q1, Q3)	67.13 (56.32, 77.23)	67.12 (56.24, 77.04)	67.3 (57.02, 78.79)	0.221
Sex, n (%)				0.061
Female	3107 (42)	2764 (42)	343 (46)	
Male	4231 (58)	3822 (58)	409 (54)	
Ethnicity, n (%)				< 0.001
White	4857 (66)	4394 (67)	463 (62)	
Black	912 (12)	780 (12)	132 (18)	
Asian	311 (4)	283 (4)	28 (4)	
Other	1258 (17)	1129 (17)	129 (17)	
SOFA, median (Q1, Q3)	7 (5, 10)	7 (5, 10)	7 (5, 10)	0.912
GCS, median (Q1, Q3)	15 (13, 15)	15 (13, 15)	15 (13, 15)	0.668
LOS hospital, median (Q1, Q3)	17.25 (9.79, 29.12)	16.89 (9.54, 28.66)	21.57 (12.29, 34.94)	< 0.001
LOS ICU, median (Q1, Q3)	6.27 (2.96, 12.78)	6.22 (2.94, 12.74)	6.81 (3.25, 13.2)	0.06
pH, median (Q1, Q3)	7.42 (7.37, 7.46)	7.42 (7.37, 7.46)	7.42 (7.37, 7.46)	0.966
Platelets, median (Q1, Q3)	207 (135, 292)	206 (135, 290)	216 (138.75, 311)	0.059
WBC median (Q1, Q3)	14.3 (9.7, 20.5)	14.2 (9.7, 20.3)	14.95 (9.97, 21.6)	0.063
Anion gap, median (Q1,Q3)	18 (15, 21)	18 (15, 21)	18 (15, 21)	0.407
BUN, median (Q1, Q3)	33 (21, 54)	33 (21, 53)	34 (21, 56.25)	0.356
Creatinine, median (Q1, Q3)	1.7 (1.1, 3.1)	1.7 (1.1, 3)	1.8 (1.1, 3.5)	0.186
PT, median (Q1, Q3)	16.1 (13.6, 21.6)	16.1 (13.6, 21.7)	15.7 (13.4, 21)	0.094
PTT, median (Q1, Q3)	37.1 (30.2, 55.5)	37.1 (30.2, 55.5)	36.9 (30.8, 55.2)	0.555
ALT median (Q1, Q3)	28 (16, 64)	29 (17, 65)	26 (15, 60)	0.033
ALP, median (Q1, Q3)	94 (66, 146)	93 (66, 145)	98 (68, 159)	0.006
AST, median (Q1, Q3)	47 (27, 108)	47 (27, 109)	44 (26, 96)	0.074
Heart rate, median (Q1, Q3)	88.71 (77.82, 101.42)	88.55 (77.63, 101.22)	91.03 (79.15, 104.05)	0.008
Systolic blood Pressure, median (Q1, Q3)	110.87 (102.73, 122.42)	111.07 (102.85, 122.51)	109.73 (101.64, 121.56)	0.025
Diastolic blood pressure, median (Q1, Q3)	59.32 (53.08, 66.12)	59.31 (53.04, 66.12)	59.38 (53.35, 65.97)	0.949
Mean blood pressure, median (Q1, Q3)	73.83 (68, 80.83)	73.9 (68.04, 80.88)	73.02 (67.76, 80.1)	0.16
Respiratory rate, median (Q1, Q3)	19.92 (17.29, 23.2)	19.92 (17.3, 23.21)	19.93 (17.17, 23.08)	0.908
Temperature, median (Q1, Q3)	36.84 (36.56, 37.21)	36.84 (36.56, 37.21)	36.84 (36.55, 37.17)	0.552
Spo2, median (Q1, Q3)	97.19 (95.69, 98.6)	97.17 (95.68, 98.59)	97.41 (95.71, 98.63)	0.297
Glucose, median (Q1, Q3)	137.43 (113.6, 173.43)	137.25 (113.67, 173.5)	139 (111.71, 171.89)	0.96
Urine output, Median (Q1, Q3)	1040 (410, 1824)	1050 (425, 1840)	936 (285.75, 1750)	0.001
Ventilation, n (%)				0.053
No	2295 (31)	2036 (31)	259 (34)	
Yes	5043 (69)	4550 (69)	493 (66)	
Vasoactive, n (%)				0.097
No	2686 (37)	2432 (37)	254 (34)	
Yes	4652 (63)	4154 (63)	498 (66)	
RRT, n (%)				0.074
No	6079 (83)	5474 (83)	605 (80)	
Yes	1259 (17)	1112 (17)	147 (20)	
Source infection				0.853
Fungi, n (%)				
No	6024 (82)	5409 (82)	615 (82)	
Yes	1314 (18)	1177 (18)	137 (18)	
Gram negative, n (%)				0.014
No	6049 (82)	5454 (83)	595 (79)	
Yes	1289 (18)	1132 (17)	157 (21)	
Gram positive, n (%)				< 0.001
No	5740 (78)	5315 (81)	425 (57)	
Yes	1598 (22)	1271 (19)	327 (43)	
Virus, n (%)				0.21
No	7294 (99)	6549 (99)	745 (99)	
Yes	44 (1)	37 (1)	7 (1)	
Original of infection, n (%)				0.034
Blood	1829 (25)	1653 (25)	176 (23)	
Bone marrow	3 (0)	3 (0)	0 (0)	
Digestive system	445 (6)	390 (6)	55 (7)	
Genitourinary system	1210 (16)	1079 (16)	131 (17)	
Immune system	58 (1)	46 (1)	12 (2)	
Joint	8 (0)	5 (0)	3 (0)	
Nervous System	42 (1)	34 (1)	8 (1)	
Parasite	6 (0)	5 (0)	1 (0)	
Respiratory system	3360 (46)	3031 (46)	329 (44)	
Skin	152 (2)	137 (2)	15 (2)	
Tumor	3 (0)	3 (0)	0 (0)	
Other organ	222 (3)	200 (3)	22 (3)	
Duration from Admission to stool findings, median (Q1,Q3)	8.16 (3.79, 13.66)	8.36 (4, 13.73)	6.47 (1.89, 13.01)	< 0.001
AKI, n (%)				0.093
I	3696 (50)	3343 (51)	353 (47)	
II	1295 (18)	1161 (18)	134 (18)	
III	2347 (32)	2082 (32)	265 (35)	
Hospital survival, n (%)				0.04
Survival	5500 (75)	4960 (75)	540 (72)	
Dead	1838 (25)	1626 (25)	212 (28)	
90-day survival, n (%)				0.022
Survival	3749 (51)	3395 (52)	354 (47)	
Dead	3589 (49)	3191 (48)	398 (53)	

SOFA, Sequential organ failure assessment score; GCS, Glasgow coma scale; LOS, Length of Stay; ALT, Alanine transaminase; ALP, Alkaline phosphatase; AST, Aspartate aminotransferase; Spo2, Oxygen saturation. RRT, renal replacement therapy.

**Table 2:** Microorganisms in stool specimens.

	Type of organism	Number
Viruses	Adenoviridae	6
	Enterovirus	1
Enterobacteriaceae	Salmonella enteritidis	2
	Salmonella species	4
Enterococcus	Enterococcus faecalis	5
Other bacteria	Campylobacter jejuni	2
	Clostridioides difficile	730
	Methicillin-resistant Staphylococcus aureus (MRSA)	1
Protists	Chilomastix mesnili	1
	Blastocystis spp	2
	Giardia intestinalis	2

### The impact of PPS on sepsis AKI

There was no statistical difference between the SOFA and GCS scores, indicating a similar level of critical illness in both groups of patients (Table 1, *P* > 0.05). The median length of hospital stays for the NPS and PPS groups was 17 days (range: 10–29) and 22 days (range: 12–35), respectively (Table 1, *P* < 0.001). In-hospital mortality was calculated using the chi-squared test and showed a statistically significant difference (Table 1, *P* = 0.04). The mortality rate for patients with a positive stool test was 28% compared to 25% for patients with a negative stool test. There was no statistically significant difference observed between the white blood cells (WBC) and platelets (Table 1, *P* > 0.05). There was also no statistical difference in the number of using RRT treatment between the two groups (Table 1, *P* > 0.05). Regarding the sources of infectious pathogens, there are certain statistical differences between gram-negative bacteria and gram-positive bacteria. There were statistical differences in the source of infection, but most of the infections in the two groups occurred in the respiratory and blood systems (Table1, *P* < 0.05). There was a statistically significant difference in the time it took to test for fecal pathogens between the two groups, with the PPS group taking an average of 6 days and the NPS group taking an average of 8 days (Table1, *P* < 0.001).

### Impact of PPS on 90-Day survival, hospital survival, and 30-day survival

**Figure 1:**
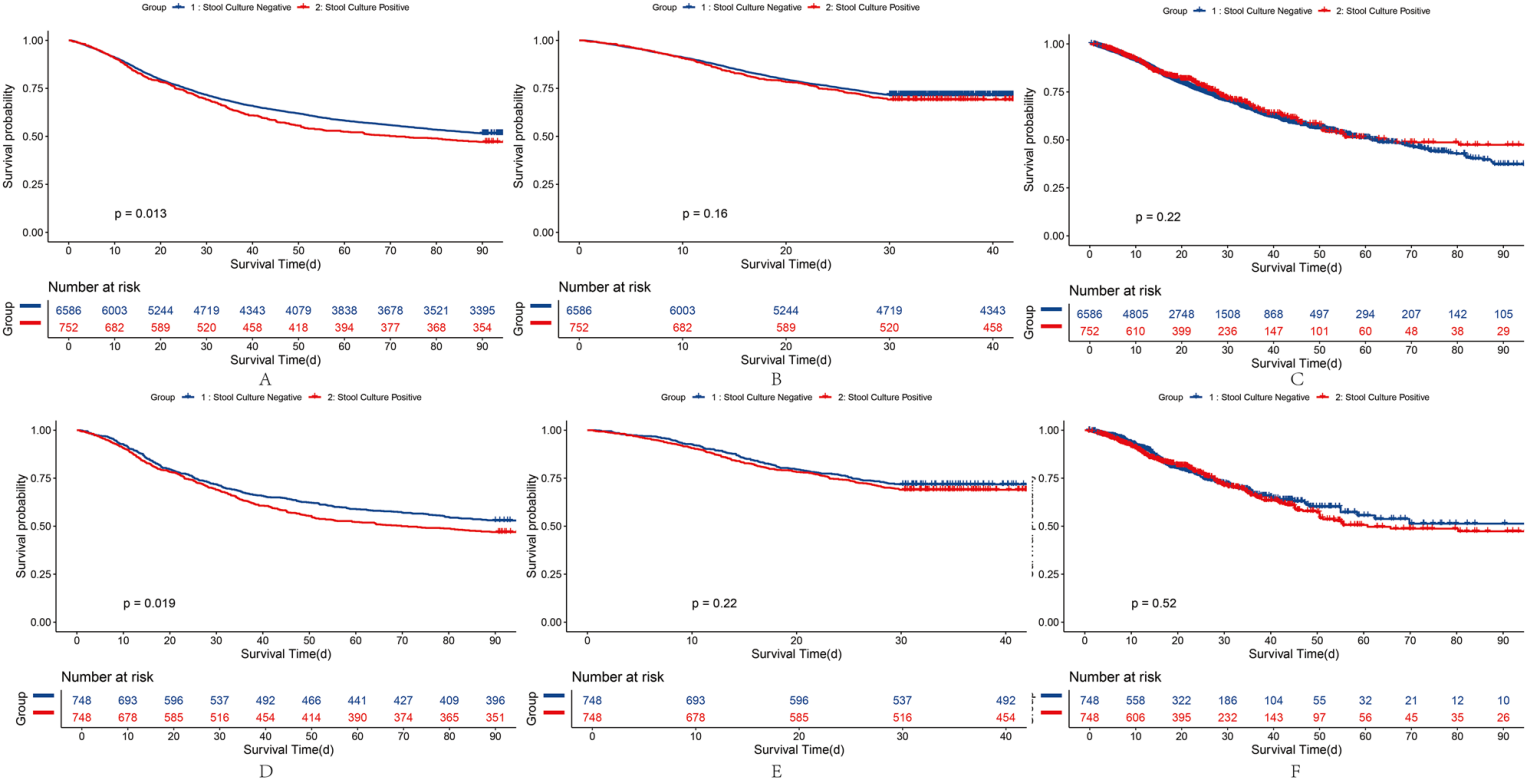
KM Curve and after-PSM KM Curve Plot of sepsis-associated AKI patients on the basis of the results of stool cultures. A, D, 90 days survival of raw data and after PSM; B, E,30 days survival of raw data and after PSM; C, F, Hospital Survival of raw data and after PSM.

**Figure 2:**
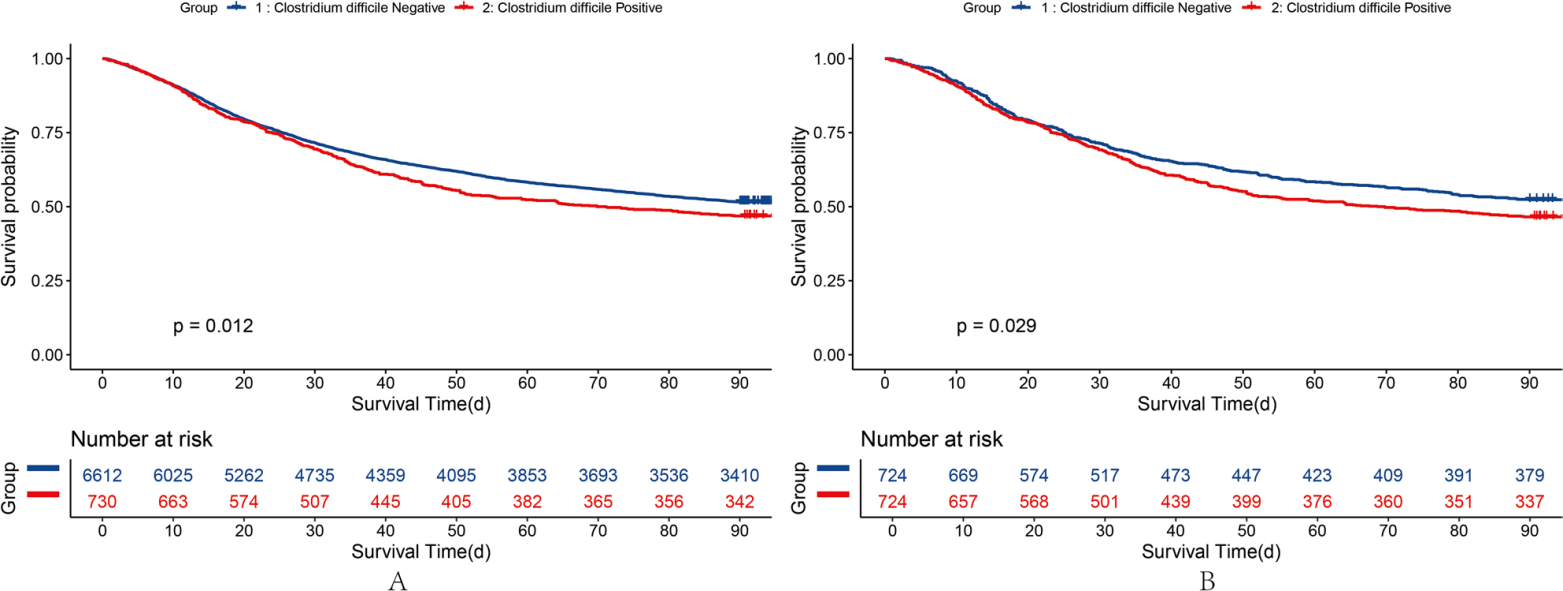
KM Curve of raw data and after PSM of 90 days Survival of sepsis-associated AKI patients on the basis of the results of *C. difficile*.

The KM curves demonstrate that mortality in patients with sepsis-associated AKI was similar between the NPS (48.45%) and PPS (52.93%) groups (*P* = 0.01; Fig. 1A). The 30-day cumulative mortality rate in the PPS group was 30.85%, which was higher than the 28.35% in the NPS group (*P* = 0.16; Fig. 1B). The probability of death during hospitalization in the PPS group (42.25%) was slightly higher than in the NPS group (43.69%; *P* = 0.21; Fig. 1C). The KM curves after PSM of hospital survival, 30-day survival, and 90-day survival are presented in Fig. 1D–F. Differences between the two groups after PSM did not alter our results. Enterobacteriaceae and Enterococci in stool samples were not associated with patient survival during hospitalization or with 30-day and 90-day survival. However, the presence of *C. difficile* in stool samples was significantly associated with decreased 90 days survival (Fig. 2A,B, Table 3). We performed causal inference of *C. difficile* positivity and 90-day prognosis, removed confounders, and drew a DAG diagram (Supplementary Fig. 1). After PSM analysis, the average treatment effect was 0.05 (95% CI 0.01–0.08, *P* < 0.05). This means that patients who develop *C. difficile* stool positivity have a 5% lower 90-day survival rate than those whose stool without *C. difficile*.

**Table 3:** Effect of PPSs on 90 days, 30 days and hospital survival.

Characteristics	90 days survival			Hospital survival			30 days survival		
	Hazard Ratio	95%CI	*P* Value	Hazard Ratio	95%CI	*P* Value	Hazard Ratio	95%CI	*P* Value
Enterobacteriaceae	0.28	0.04–2.01	0.207	0	0-Inf	0.975	0.54	0.08–3.82	0.536
Enterococcus	0.91	0.23–3.64	0.895	0.75	0.11–5.31	0.772	1.68	0.42–6.71	0.465
Other bacteria	1.14	1.03–1.27	0.013	0.91	0.79–1.06	0.221	1.09	0.95–1.25	0.23
Viruses	0.86	0.28–2.66	0.792	0.69	0.17–2.74	0.594	1.01	0.25–4.05	0.988
Protists	2.83	1.06–7.55	0.037	5.27	1.97–14.06	0.001	3.71	1.39–9.91	0.009

CI, confidence interval; HR, hazard ratio.

Variables that showed differences in 90-day patient survival were analyzed using single-factor Cox analysis with stool culture. Supplementary Table 1 demonstrates all variables initially considered in the forward stepwise model. The correlation between these factors and survival was statistically significant (Table 4).

**Table 4:** Multivariate analysis of risk factors of 90 days Survival.

Variables	Hazard ratio	95% CI	*P* Value
Lactate	1.07	1.05–1.09	< 0.001
Vasoactive	1.38	1.28–1.50	< 0.001
Duration from admission to stool findings	0.96	0.96–0.97	< 0.001
Total bilirubin	1.01	1.01–1.02	< 0.001
Vancomycin	1.36	1.24–1.5	< 0.001
Other antibiotics	1.23	1.15–1.33	< 0.001
AKI	1.16	1.11–1.21	< 0.001
Creatinine	0.9	0.88–0.93	< 0.001
BUN	1.01	1.00–1.01	< 0.001
Neutrophils abs	1.01	1.01–1.01	< 0.001
Spo2	0.99	0.99–0.99	< 0.001
Metronidazole	1.19	1.11–1.28	< 0.001
Diabetes without complications	0.9	0.83–0.97	0.006
Quinolones	0.83	0.77–0.90	< 0.001
Cerebrovascular disease	1.44	1.32–1.58	< 0.001
Liver	1.07	1.03–1.12	0.002
Age	1.02	1.01–1.02	< 0.001
Metastatic solid tumor	1.97	1.72–2.25	< 0.001
Ethnicity	1.08	1.05–1.12	< 0.001
Severe liver disease	1.38	1.21–1.57	< 0.001
Aminoglycosides	1.43	1.25–1.64	< 0.001
Malignant cancer	1.32	1.2–1.46	< 0.001
Total co2	1.02	1.01–1.02	< 0.001
Mean blood pressure	0.99	0.99–1.00	< 0.001
fungi infection	1.18	1.09–1.29	< 0.001
Myocardial infarct	1.22	1.12–1.32	< 0.001
Temperature	0.9	0.85–0.95	< 0.001
Anion gap	1.02	1.01–1.03	0.001
Sulfonamides	0.82	0.72–0.93	0.002
INR	1.07	1.02–1.12	0.002
Mild liver disease	1.15	1.04–1.27	0.006
Verbal of GCS	0.97	0.95–0.99	0.007
Coagulation score of SOFA	1.04	1.01–1.08	0.022

Because treatment is already in using at the time of stool examination, antibiotics have an effect on fecal pathogens. The use of antibiotics was described in both groups (Table 5). In the group with positive pathogenic bacteria, vancomycin was used more frequently, with about 88% of patients using it, and there was a statistical difference between the two groups. Similarly, metronidazole was used more often in the PPS group and less often in the NPS group (*P* < 0.001). Penicillin/cephalosporin and quinolones were used less in the PPS group than in the NPS group (*P* < 0.05). There was no statistical significance in the antibiotic use of macrolides, sulfonamides, tetracyclines and aminoglycosides between the two groups (*P* > 0.05).

**Table 5:** Antibiotics use in PPS group and NPS group.

Variables	Total (n = 7338)	NPS (n = 6586)	PPS (n = 752)	*P* value
Penicillin/Cephalosporin, n (%)				0.004
No	1227 (17)	1073 (16)	154 (20)	
Yes	6111 (83)	5513 (84)	598 (80)	
Quinolones, n (%)				< 0.001
No	5267 (72)	4686 (71)	581 (77)	
Yes	2071 (28)	1900 (29)	171 (23)	
Macrolides, n (%)				0.125
No	6393 (87)	5724 (87)	669 (89)	
Yes	945 (13)	862 (13)	83 (11)	
Sulfonamides, n (%)				0.993
No	6680 (91)	5996 (91)	684 (91)	
Yes	658 (9)	590 (9)	68 (9)	
Vancomycin, n (%)				< 0.001
No	1456 (20)	1365 (21)	91 (12)	
Yes	5882 (80)	5221 (79)	661 (88)	
Metronidazole, n (%)				< 0.001
No	4825 (66)	4530 (69)	295 (39)	
Yes	2513 (34)	2056 (31)	457 (61)	
Tetracyclines, n (%)				0.408
No	6965 (95)	6246 (95)	719 (96)	
Yes	373 (5)	340 (5)	33 (4)	
Aminoglycosides, n (%)				0.994
No	6953 (95)	6241 (95)	712 (95)	
Yes	385 (5)	345 (5)	40 (5)	
Others antibiotic, n (%)				0.754
No	5203 (71)	4674 (71)	529 (70)	
Yes	2135 (29)	1912 (29)	223 (30)	

## Discussion

In this study, we observed that the presence of *C. difficile* protists in stool samples was associated with a higher risk of in-hospital death, 30-day death, and 90-day death in patients with sepsis-associated AKI. PPS is associated with poor survival, particularly in those with *C. difficile* in their stool samples. To our knowledge, this study provides the first opportunity to investigate the exploratory role of PPS in patients with sepsis-associated AKI.

In this study, we observed that the presence of *C. difficile* and protists in stool samples significantly increased the risk of 90-day mortality. Protists were associated with worse in-hospital and 30-day survival. Multiple stool tests are necessary to avoid false negatives, so we did not include confounders in the number of tests^17^. Taking into account prolonged hospitalization with multiple fecal examinations, a worse 90-day survival was observed in PPS patients after propensity score matching to adjust for the possible influence of confounders.

*C. difficile* and protists in stool samples is associated with sepsis association AKI, and infections, and dysbiosis may negatively impact their survival^18,19^. This phenomenon also is due to increased levels of inflammatory cytokines and the fact that ischemia can lead to changes in the constituent organisms that make up the intestinal pathogens^20,21^. Sepsis can lead to a significant loss of microbial diversity in patients^22^. In our study, patients with elevated absolute neutrophil counts had lower survival rates than patients with decreased absolute neutrophil counts. Gastrointestinal colonization with *C. difficile* may be a potential risk factor for invasive fungal in sepsis-associated AKI patients. However, common dysbiosis pathogenic bacterial infections were not found in this study, like candidiasis. Therefore, patients with sepsis-associated AKI should be monitored for the presence of *C. difficile* in stool samples.

Patients whose stool samples contained *C. difficile* had worse survival outcomes than those with persistently NPS. In patients with sepsis-associated AKI, those whose intestines are colonized with antibiotic-resistant bacteria are likely to develop bacteremia. Inhibiting the intestinal flora can significantly reduce the incidence of bacteremia. High concentrations of *C. difficile* bacteria in feces can result in a high risk of bacterial translocation into the bloodstream. Microbial abundance and diversity in hemodialysis patients are on a downward trend. In addition, the mortality rate of sepsis-associated AKI patients undergoing further kidney replacement therapy is high, reaching up to 50%^23^. Broad-spectrum antibiotics increase the risk of Clostridium difficile infection. Therefore, reducing the use of high-risk antibacterial drugs associated with Clostridium difficile infection (such as second- and third-generation broad-spectrum cephalosporins, fluoroquinolones, clindamycin, etc.), as well as their frequency and duration, may improve patient clinical outcomes^24,25^.

Protists organisms constitute a heterogeneous category of eukaryotic microbes that bear a potential association with heightened mortality in individuals suffering from sepsis-related acute kidney injury^26^. The poor prognosis may be due to the severe immune stress caused by protists^27^. For instance, protozoan infections can lead to endothelial dysfunction, hinder perturb the integrity of the glycocalyx layer as well as foster platelet aggregation and the initiation of coagulation cascades culminating in fibrin deposition^28^. Our results resonate with prior studies, affirming the contributory role of protists in augmenting patient fatality rates.

In the ICU, patients with sepsis association AKI have lower long-term 90-day survival. Our analysis showed that patients who were positive pathogens in stool had lower 90-day survival rates. This is somewhat related to treatment. Vancomycin, a potent antibiotic that primarily targets Gram-positive bacteria, is particularly effective against methicillin-resistant Staphylococcus aureus (MRSA) and other drug-resistant staphylococci^29^. In the ICU, where patients with sepsis have PPS, the increased use of vancomycin may be indicative of efforts to control *C. difficile* infections rather than association with sepsis-associated acute kidney injury (AKI). In addition, our multivariate regression showed that it was associated with 90-day survival and also associated with quinolones. Quinolones are used less frequently in patients with positive stools, which may indicate the need for caution in clinical use. The source of the fungal infection also has a negative impact on the patient's prognosis for 90-day survival. Due to the patient's compromised immune function, it is difficult for the host to eliminate fungi, toxins, and metabolites^30,31^. The antibiotics used have a certain nephrotoxicity, resulting in a worse 90-day prognosis than in normal patients.

In the context of clinical implications, the detection of *C. difficile* and protists in stool samples, which are associated with adverse survival outcomes, indicates a recommendation for routine fecal microbiota screening in these patients, particularly during the early phase of hospitalization. Enhanced testing frequency minimizes false-negative results, enabling prompt identification and intervention in cases of potential microbial imbalance. In light of the observed association between antibiotic use and PPS, clinicians may wish to exercise caution when prescribing second- and third- generation cephalosporins, fluoroquinolones, clindamycin, and other high-risk antibiotics. It may be advisable to consider shorter courses where feasible. Treatment strategies should prioritize alternatives with reduced nephrotoxicity to safeguard both renal function and the integrity of the gut microbiome. Maintaining microbial diversity and gut microbiome stability is vital to prevent bacterial translocation and secondary infections. Moreover, it is imperative to conduct large-scale, prospective, multi-center studies to validate these observations and elucidate the correlations between gut microbiota and clinical outcomes across diverse populations. These endeavors will facilitate the development of more precise therapeutic guidelines, tailored to the unique microbial profiles of patients with sepsis-associated acute kidney injury.

A limitation of this study is its retrospective design coupled with a relatively small sample of bacterial species in the PPS group. Therefore, the role of microorganisms in fecal samples from patients with sepsis-associated AKI warrants further investigation. Second, this study was conducted with patients in the United States, where bacterial prevalence may vary. This variability could potentially influence the association between fecal microorganisms and clinical outcomes in other populations. Third, retrospective studies may have inherent flaws. For example, selection bias, included patients mainly collected stool after treatment, and treatment caused different causes of stool. Prospective studies are needed to investigate the correlation between the presence of stool pathogens at admission and prognosis.

In conclusion, our results suggest that PPS significantly influence the 90 days survival outcomes of patients with sepsis association AKI, particularly those with *C. difficile* in their stool samples. Depending on the differential detection of positive and negative antibiotics in stool samples, clinicians may find it beneficial to adjust their antimicrobial therapy regimens accordingly.

## Methods

### Patients

**Figure 3:**
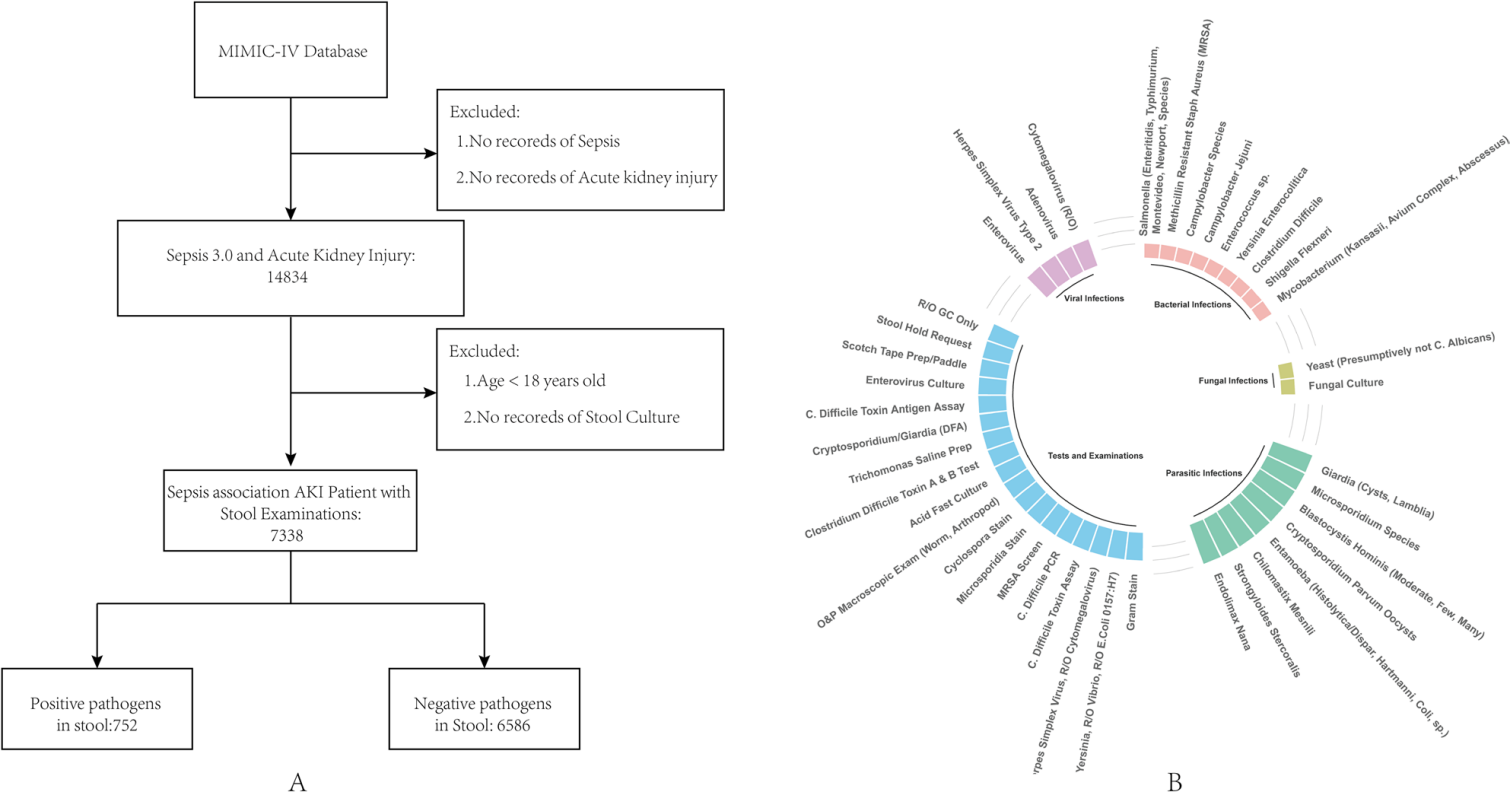
Cohort flow diagram. A, MIMIC-IV. B, Various stool pathogen tests.

This study conducted a retrospective analysis of the MIMIC database, a comprehensive resource for intensive care patients^32^. The MIMIC database was established in 2003 through a collaborative effort involving the Beth Israel Deaconess Medical Center (BIDMC), MIT, the National Institutes of Health, Massachusetts General Hospital, emergency physicians, critical care physicians, computer scientists, and other critical care professionals. The current version, MIMIC-IV, includes patients admitted between 2008 and 2019. Prior to utilizing the database, our team completed the necessary training and obtained the appropriate certifications. As this project does not impact clinical care, all sensitive health information remains anonymous, thereby eliminating the need for individual patient consent. We followed the official MIMIC-IV tutorial to construct the research database using PostgreSQL (version 10.0, PostgreSQL Global Development Group). We extracted data from patients with sepsis-associated AKI who underwent comprehensive stool culture analysis for all bacteria and fungi in the gastrointestinal tract. The exclusion criteria for this study are as follows:1) Patients who are younger than 18 years old; 2) Patients with missing AKI diagnostic information, creatinine value, or urine volume. 3) There is a lack of information regarding the diagnosis of sepsis, and there is no record of the SOFA score or infection information. 4) There are no records of stool or digital rectal examinations for the patients. A total of 7338 patients were included (Table 1). Figure 3a shows the patient screening flowchart, while Fig. 3b illustrates various stool pathogen tests.

### Definitions and assessments

Patients with sepsis were selected and diagnosed according to the Third International Consensus Definitions for Sepsis and Septic Shock (Sepsis-3)^33^. In addition, we compiled additional codes as recommended by MIMIC-IV. Acute kidney injury (AKI) was defined according to the Kidney Disease: Improving Global Outcomes (KDIGO) criteria, which specify that AKI is either an increase of 0.3 mg/dl within 48 h or a 50% increase over baseline serum creatinine (sCr)^34^. The primary endpoint was 90-day survival. Secondary endpoints included in-hospital death and 30-day survival. The definition of positive pathogens in stool, during this ICU treatment, positive pathogens found in stool, digital rectal examination, and fecal swab were defined as positive pathogens in stool.

### Statistical analysis

Missing values were imputed multiple times using the 'mice' package (version 3.15.0) in R. Baseline variables were compared using independent samples t-tests or Mann–Whitney U tests as appropriate. Survival probabilities were estimated using the Kaplan–Meier (KM) method. Potential prognostic factors were assessed by multivariate analysis using Cox proportional hazards regression and stepwise model selection methods. To illustrate the relationship between these three variables, we used a Directed Acyclic Graph (DAG). Because there were multiple stool tests for a long hospital stay and only one test for a short hospital stay, we considered length of treatment in hospital as a confounding factor and performed propensity score matching. Propensity scores were then used to control for confounding by R package ‘MatchIt’. The average treatment effect (ATE) was calculated using inverse probability weighting in this study. The multivariate Cox proportional hazards model included variables with statistically significant differences in individual factors. The ATE represents the causal effect of the variable of interest on patient 90 days causal inference radio in both the death and non-death study populations.

Independent variables with *P* > 0.05 were sequentially excluded from the model, while independent variables with *P* < 0.05 were considered statistically significant. The 'CBCgrp' package (version 2.8.2) in R (version 4.1.2) was used to calculate data^35^. All reported *P*-values are based on two-tailed hypothesis testing. Data analysis was primarily performed using competing risk analysis in the R package (version 4.1.2).

### Supplementary Information

Supplementary Figure 1.

Supplementary Table 1.

## Data Availability

The data is open source and freely available at https://www.physionet.org/content/mimiciv/2.2.
