# Supplementary figures and images for: Positive pathogens in stool could predict the clinical outcomes of sepsis-associated acute kidney injury in critical ill patient
Source: Sci Rep. 2024 May 16;14:11227. doi: 10.1038/s41598-024-62136-6 (PMC11099037; doi:10.1038/s41598-024-62136-6)

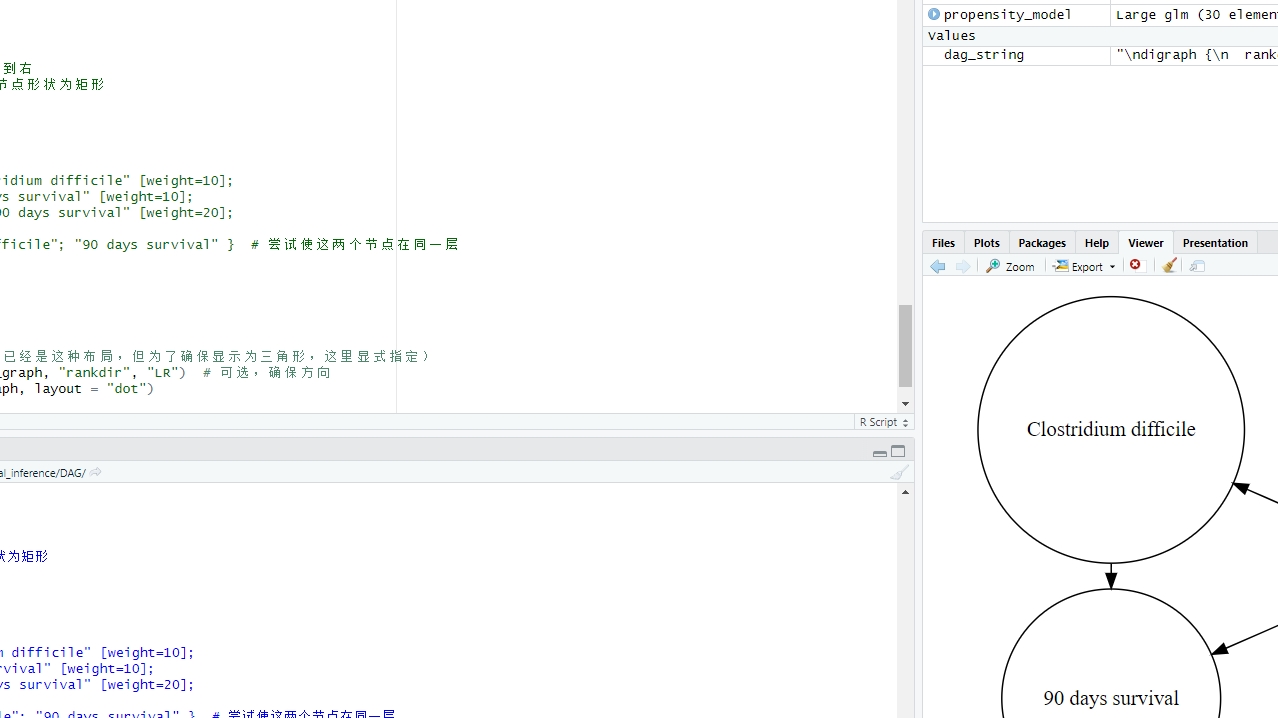

Supplement: Supplementary file 1 — Supplementary Figure 1. [file 41598_2024_62136_MOESM1_ESM.jpeg]
